# Performance of the *Aspergillus* galactomannan lateral flow assay with a digital reader for the diagnosis of invasive aspergillosis: a multicenter study

**DOI:** 10.1007/s10096-023-04724-4

**Published:** 2023-11-29

**Authors:** Jian Guo, Chenlu Xiao, Wenjie Tian, Li Lv, Liang Hu, Lijun Ni, Dongjiang Wang, Wei Li, Dan Qiao, Wenjuan Wu

**Affiliations:** 1 Department of Laboratory Medicine, Shanghai East Hospital, Tongji University School of Medicine, Shanghai, China; 2 Department of Laboratory Medicine, Ruijin Hospital Affiliated to Shanghai Jiao Tong University School of Medicine, Shanghai, China; 3 Department of Clinical Microbiology, Ruijin Hospital Affiliated to Shanghai Jiaotong University School of Medicine, Shanghai, China; 4 Department of Clinical Laboratory, Qilu Hospital of Shandong University, Jinan, China

**Keywords:** Invasive aspergillosis, Aspergillus galactomannan, Lateral flow assay, Sensitivity, Specificity

## Abstract

**Purpose:**

The objective of this multicenter study was to compare the diagnostic performance of lateral flow assay (LFA) and enzyme-linked immunosorbent assay (ELISA) to detect the Dynamiker *Aspergillus* Galactomannan levels in serum and bronchoalveolar lavage fluid (BALF) samples for I.

**Methods:**

We registered 310 clinically suspected *Aspergillus* infection patients from December 2021 to February 2023 and classified them into subgroups as the “IA group” and “non-IA group” based on the latest EORTC/MSG guidelines. The immunoassays were analyzed by LFA and ELISA respectively.

**Results:**

Galactomannan was examined using LFA, and serum and BALF samples demonstrated sensitivities of 82.57% and 89.47%, specificities of 90.76% and 92.00%, PPVs of 89.11% and 96.23%, and NPVs of 85.04% and 79.31%, respectively. Galactomannan was observed using two assays in serum and BALF samples and showed PPAs of 95.11% and 93.33%, NPAs of 89.19% and 96.30%, and TPAs of 92.47% and 94.25%, respectively. The ROC curve demonstrated that LFA had optimum diagnostic value when the index value (*I* value) = 0.5, the sensitivity was 84.94%, and the specificity was 90.97%.

**Conclusion:**

Compared to the ELISA method, the LFA has shown excellent performance for the diagnosis of IA in serum and BALF sample and can be used as an assay for the early diagnosis of patients with IA. The dynamic change in galactomannan levels may be useful for assessing treatment response.

## Introduction

According to cutting-edge research by Fudan University in Shanghai, China, and the Global Action Fund for Fungal Infections (GAFFI), as many as 300,000 people worldwide are affected by invasive aspergillosis (IA), and currently, an estimated 1,179,000 people are affected by IA in China alone. This study did not include COVID-19 and influenza cases, two additional risk factors for IA [1, 2].

People at risk of IA and chronic aspergillosis included 774,000 patients with lung cancer; 29,000,000 patients hospitalized for chronic obstructive pulmonary diseases (COPDs) such as emphysema; 844,000 patients with pulmonary tuberculosis (PTB); 41,200 patients with acute myelogenous leukemia; 106,000 patients with advanced AIDS; and 21,400 transplant recipients[3, 4]. The incidence of IA is very high in patients with severe influenza and COVID-19, but it is difficult to estimate the incidence because the number of affected patients varies from year to year. IA is a leading cause of death in acute leukemia and bone marrow transplant patients. Timely initiation of antifungal therapy can determine life or death in these patients.

*Aspergillus* galactomannan is a polysaccharide component widely found in the cell wall of *Aspergillus*. After *Aspergillus* invades lung tissue, early galactomannan is released in body fluids and blood, such as BALF, cerebrospinal fluid, and pleural fluid. Galactomannan levels can be tested in blood or body fluids before clinical symptoms and signs appear in IA patients, so the galactomannan test is a prominent tool in the early diagnosis of IA[5, 6]. The most frequently used method for antigen detection is the enzyme-linked immunosorbent assay (ELISA), the results of which can be reported within 2 h. In the EORTC/MSG guidelines, galactomannan tests are advocated for early and rapid diagnosis [7].

Several new approaches have also been developed, one of which is the LFA. An LFA is an independent immunochromatographic assay similar to a home pregnancy test for the qualitative and semiquantitative testing of galactomannan in serum and BALF samples; the test produces results in approximately 20 min and has good concordance with ELISA [8, 9]. Early and fast diagnosis of IA followed by targeted antifungal therapy has the potential to significantly improve survival. Continuous galactomannan testing is an option for IA testing in these patients, and ELISA and LFA commercial kits have been validated in some clinical studies [10].

The objective of this multicenter study was to determine the effectiveness of a novel QuicGM™ *Aspergillus* galactomannan Ag LFA produced by Dynamiker Biotechnology (Tianjin) Co., Ltd., used as a screening test for IA. QuicGM™ LFA is a fluorescent immunochromatographic test kit using monoclonal antibodies against galactomannan and europium nanoparticles. It is a semiquantitative assay combined with a portable detection device that can be widely accepted by the clinical and primary medical communities for the early and fast diagnosis of IA. A comparative study of LFA and ELISA was performed to validate the performance of this new assay.

## Materials and methods

### Study design and population

We organized a prospective multicenter cohort study at three medical centers, including Ruijin Hospital affiliated with the Shanghai Jiao Tong University School of Medicine, Shanghai East Hospital affiliated with the Tongji University School of Medicine, and Qilu Hospital of Shandong University. We registered 310 patients with clinically suspected *Aspergillus* infection from December 2021 to February 2023. The EORTC/MSG has developed definitions for the diagnosis of possible, probable, and proven IA. These definitions depend on host criteria (immunosuppressive conditions), clinical criteria (clinical and radiological signs of IPA), and mycological criteria (results of direct or indirect microbiological testing for *Aspergillus* species). Patients who did not conform to the definition were defined as non-IA. Ruijin Hospital approved this study (code RJ2022246) before commencement.

### LFA and ELISA analysis

Two immunoassays were used for the semiquantitative testing of *Aspergillus* galactomannan antigen, including the Dynamiker QuicGM™ *Aspergillus* galactomannan lateral flow assay (LFA) and the Dynamiker *Aspergillus* Galactomannan enzyme-linked immunosorbent assay (ELISA). According to the manufacturer’s instructions, a serum/BALF sample *I* value ≥ 0.5 was considered a positive result for LFA and ELISA.

For the LFA, 300 μL serum/BALF samples were pretreated by the addition of 100 μL sample treatment solution in 1.5-mL screw-cap polypropylene tubes, vortexed for 10 s, and centrifuged at 10,000 × g for 15 s to shake off the sample of the centrifuge tube. Depending on the laboratory apparatus, the sample was heated for 5 min in a water bath or heat block and then centrifuged at 10,000 × g for 10 min. A total of 90–100 μL of sample supernatant was transferred into the sample well. The *I* value results were recorded with the fluorescence immunoassay analyzer provided by the manufacturer after 20 min.

**Fig. 1:**
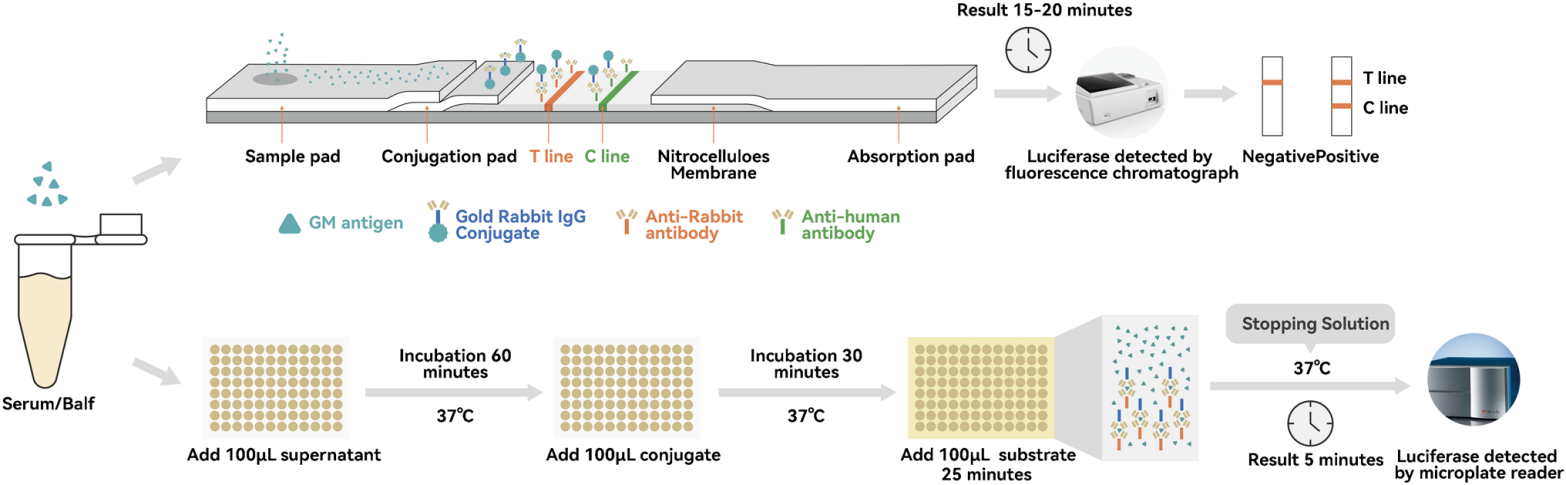
Mechanistic illustration of LFA/ELISA and representative examples of the results

For ELISA, the sample pretreatment method was consistent with that for the LFA. Then, 100 μL of supernatant was added to microtiter wells, incubated at 37 °C for 60 min, and washed. Next, 100 μL of the conjugate was added to microtiter wells, incubated at 37 °C for 30 min, and washed. Then, 100 μL of substrate solution was added to microtiter wells and incubated at 37 °C for 25 min. The stopping solution was added to microtiter wells and incubated at 37 °C for 5 min to terminate the reaction. The OD at 450 nm (reference 620/630 nm) was read within 5 min after the addition of the stopping solution (Fig. 1).

### Statistical analyses

The clinical performance of the two assays was validated in the studied population by calculating the sensitivity, specificity, positive predictive value, and negative predictive value. ROC curve analysis was used to determine the overall performance in the studied population and the best positive threshold to improve the specificity and sensitivity of the two assays. Spearman’s rank correlation coefficient was used to determine the quantitative agreement between the two assays. The Mann–Whitney test in paired analysis and the Kruskal–Wallis test were used to compare multiple median values for different groups. Statistics were performed by using GraphPad Prism 8.0.2, and *P* < 0.05 was considered significant.

## Results

### Patient characteristics

In total, 419 samples from 310 patients (166 patients in the proven/probable IA group, 144 patients in the non-IA group) were tested, including 332 serum samples and 87 BALF samples.

The patient characteristics and underlying diseases in this study are summarized in Table 1. Compared with the non-IA group, the number of males in the IA group was higher, but the difference was not statistically significant (*P* > 0.01). The non-IA group was older than the IA group (*P* < 0.01). The common underlying diseases in IA patients were diabetes mellitus, sepsis, and acute myeloid leukemia. The most common symptoms in IA patients were cough, sputum, and fever. Chest pain, dyspnea, and hemoptysis were not common in IA patients.

**Table 1:** Characteristics of the IA group and non-IA group

Characteristic	IA group *n*=166	Non-IA group *n*=144	*P*-value
Age, years (median [IQR])	65 [52,71]	69 [57,77]	0.0048
Male (%)	109 (65.7)	86 (59.7)	0.2910
Underlying disease (%)			
Diabetes mellitus	36 (21.7)	26 (18.1)	0.4777
Sepsis	19 (11.4)	6 (4.2)	0.0213
COPD	10 (6.0)	17 (11.8)	0.1048
Acute myeloid leukemia	19 (11.4)	0	<0.0001
Lymphoma	11 (6.6)	2 (1.4)	0.0239
HSCT	11 (6.6)	0	0.0011
PTB	2 (1.2)	7 (4.9)	0.0870
Acute lymphoblastic leukemia	9 (5.4)	0	0.0041
Interstitial lung disease	5 (3.0)	5 (3.5)	0.9999
Myelodysplastic syndrome	4 (2.4)	0	0.1264
Systemic symptoms (%)			
Cough	45 (27.1)	66 (45.8)	0.0008
Sputum	45 (27.1)	62 (43.1)	0.0040
Fever	30 (18.1)	32 (22.2)	0.3947
Dyspnea	4 (2.4)	9 (6.3)	0.1532
Hemoptysis	3 (1.8)	4 (2.8)	0.7084
Chest pain	3 (1.8)	7 (4.9)	0.1969
Chest CT findings (%)			
Nodules	75 (45.2)	46 (31.9)	0.0197
Pleural effusion	45 (27.1)	33 (22.9)	0.4325
Consolidation	9 (5.4)	5 (3.5)	0.5851
Bronchiectasis	8 (4.8)	4 (2.8)	0.3932
Cavitation	8 (4.8)	6 (4.2)	0.9999
Fungal culture (%)			
*A. fumigatus*	7 (4.2)	-	-
*A. terreus*	1 (0.6)	-	-
*A. nidulans*	1 (0.6)	-	-
mNGS (%)			
*A. fumigatus*	6 (3.6)	-	-
*A. terreus*	2 (1.2)	-	-
*A. flavus*	1 (0.6)	-	-
Neutropenia (%)	17 (10.2)	6 (4.2)	0.0506
Agranulocytosis (%)	8 (4.8)	0	0.0082
Overall mortality (%)	19 (11.4)	14 (9.7)	0.7132
Serum GM ODI (median [IQR])	1.05 [0.58, 1.79]	0.34 [0.26, 0.38]	<0.0001
BALF GM ODI (median [IQR])	1.30 [0.61, 2.33]	0.43 [0.39, 0.47]	0.0022

*COPD*, chronic obstructive pulmonary disease; *HSCT*, hematopoietic stem cell transplantation; *PTB*, pulmonary tuberculosis; *mNGS*, metagenomic next-generation sequencing; *IQR*, interquartile range

**Fig. 2:**
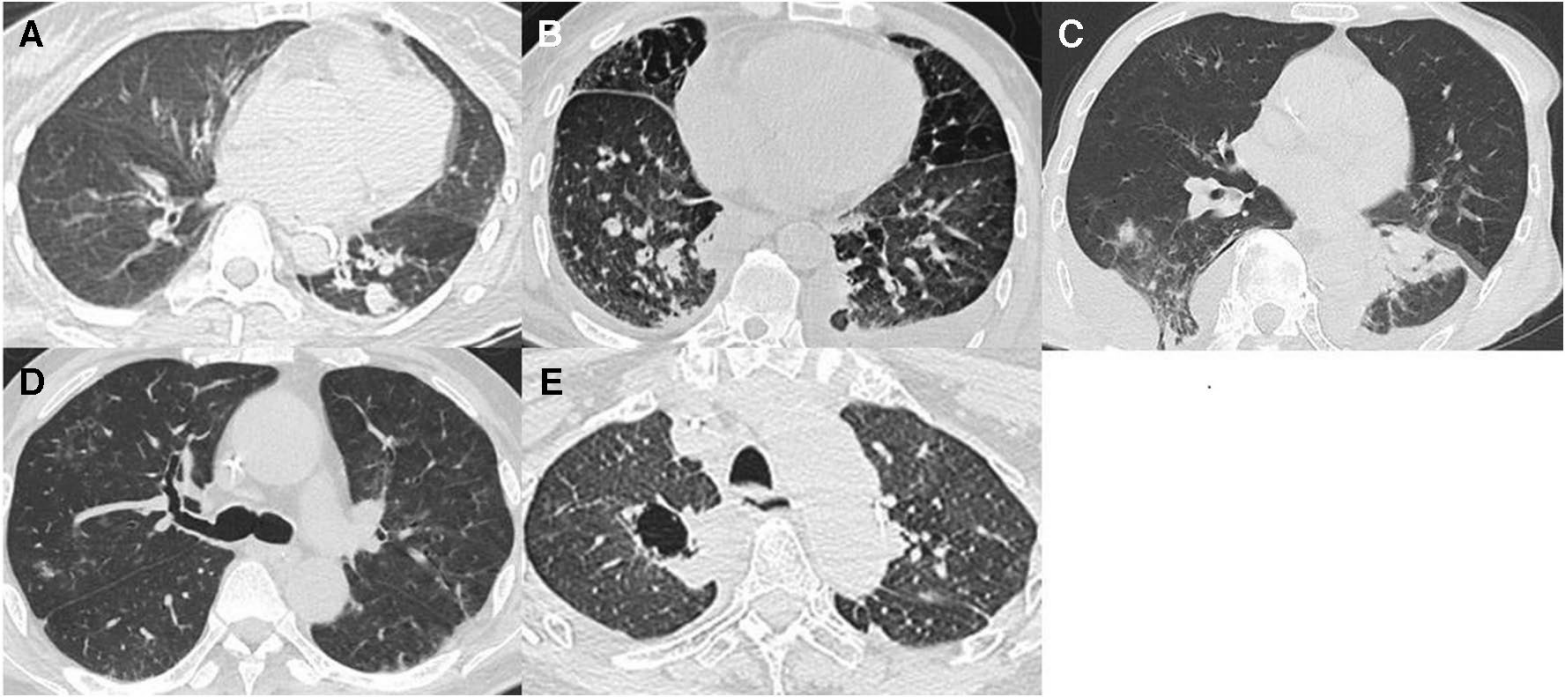
Representative examples of chest CT findings in IA patients. **A** Aspergillus nodules in the right upper and lower lobes and left lower lobe. **B** An Aspergillus nodule with pleural effusion in the left lobe. **C** Consolidation in the right middle lobe and left upper lobe. **D** Bronchiectasis in the right lower and middle lobes. **E** An Aspergillus nodule with cavitary lesions in the right middle lobe.

Fig. 2 shows representative chest computed tomography (CT) images of some cases. In these IA patients, specific chest CT findings, such as the air crescent sign and halo sign, were rare. The chest CT findings showed that the incidence of single or multiple nodules was consistent with other reports [11].

*Aspergillus* spp. were isolated from eight patients. One of the patients was infected with both *A. terreus* and *A. nidulans*. *Aspergillus* spp. were tested in seven patients by mNGS. One of the patients was infected with *A. fumigatus*, *A. terreus*, and *A. flavus*. The differences in the median GM *I* value in the serum and BALF samples between the IA group and the non-IA group were significant (*P* < 0.0001 and 0.0022).

**Fig. 3:**
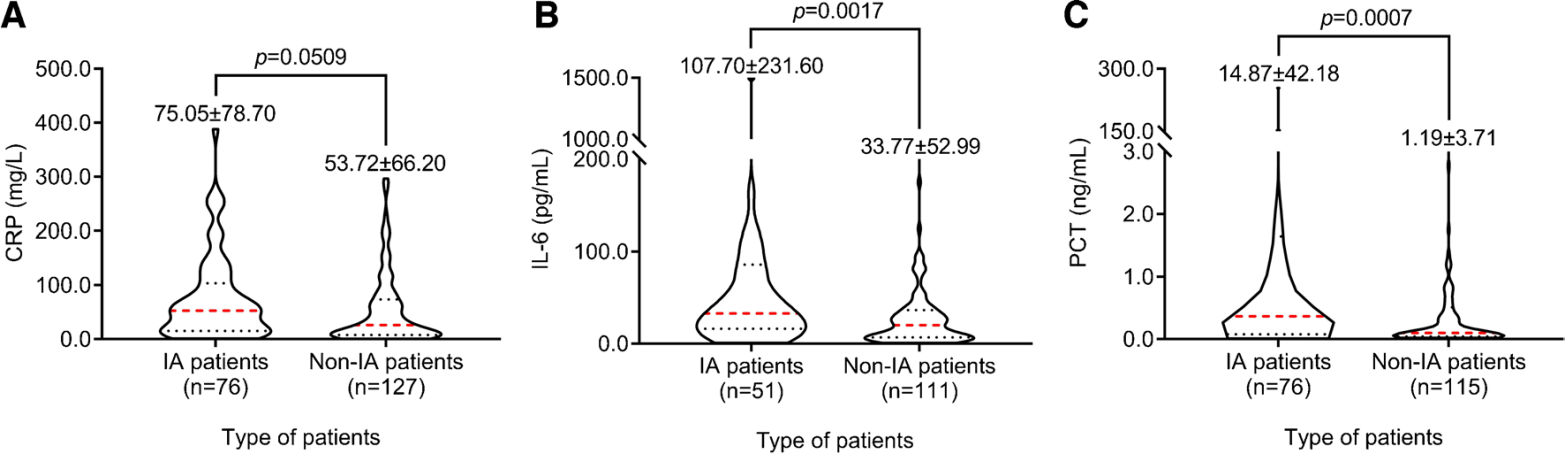
Comparisons of CRP concentration (**A**), IL-6 concentration (**B**), and PCT concentration (**C**) in peripheral blood between IA and non-IA patients. The mean and standard error are shown in the figures.

There was no significant difference in the C-reactive protein (CRP) concentration in peripheral blood between the IA group and the non-IA group (*P* > 0.05). The IA group showed significantly higher levels of interleukin-6 (IL-6) and procalcitonin (PCT) in peripheral blood than the non-IA group (*P* < 0.05, Fig. 3). Previous studies confirmed that IL-6 and PCT concentrations were positively correlated with galactomannan levels. This result suggests that the diagnostic potential of more cytokines for IA should be tested, and their binding with other IA biomarkers should be evaluated [12–14].

### Percent agreement of LFA and ELISA

In serum sample, BALF samples, and total samples, the total percent agreement (TPA) between LFA and ELISA was 92.47%, 94.25%, and 92.84%, respectively, representing good agreement.

The positive percent agreement (PPA) and negative percent agreement (NPA) for serum samples were 95.11% and 89.19%, respectively, with most of the discordance arising due to samples that were negative by LFA but positive by ELISA. The PPA and NPA for BALF samples were 93.33% and 96.30% (Table 2), respectively.

**Table 2:** Observed qualitative sample agreement between LFA and ELISA

Sample type	PPA (95% CI)	NPA (95% CI)	TPA (95% CI)	Kappa
Serum (*n*=332)	95.11 (175/184) 74.46–85.34	89.19 (132/148) 67.23–82.44	92.47 (307/332) 93.10–95.60	0.85
BALF (*n*=87)	93.33 (56/60) 56.15–83.04	96.30 (26/27) 42.31–85.99	94.25 (82/87) 85.00–93.67	0.87
Total (*n*=419)	94.67 (231/244) 72.85–83.14	90.29 (158/175) 72.88–84.84	92.84 (389/419) 92.85–95.20	0.85

### Concordance between LFA and ELISA

**Fig. 4:**
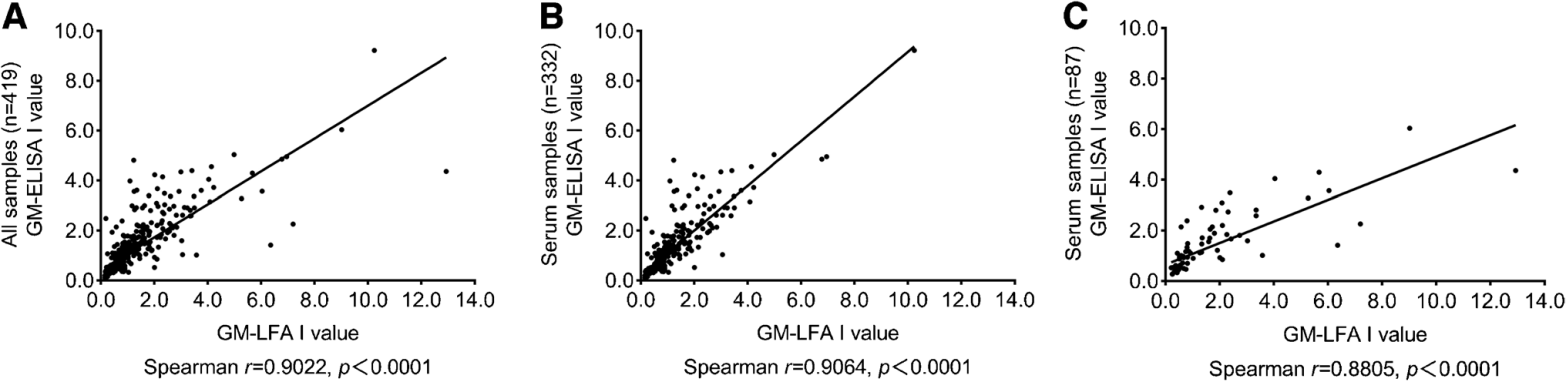
Linear correlation between the GM *I* value generated by LFA and ELISA when testing all samples (**A**), serum samples (**B**), and BALF samples (**C**).

The semiquantitative correlation between the GM *I* values calculated by LFA and ELISA was excellent (Spearman’s coefficient, *r* = 0.9022). For serum samples, the semiquantitative correlation between the GM *I* values for LFA and ELISA was better than that for BALF samples (Spearman’s coefficient, *r* = 0.9064 vs. *r* = 0.8805, Fig. 4).

### Analysis of detection levels

**Fig. 5:**
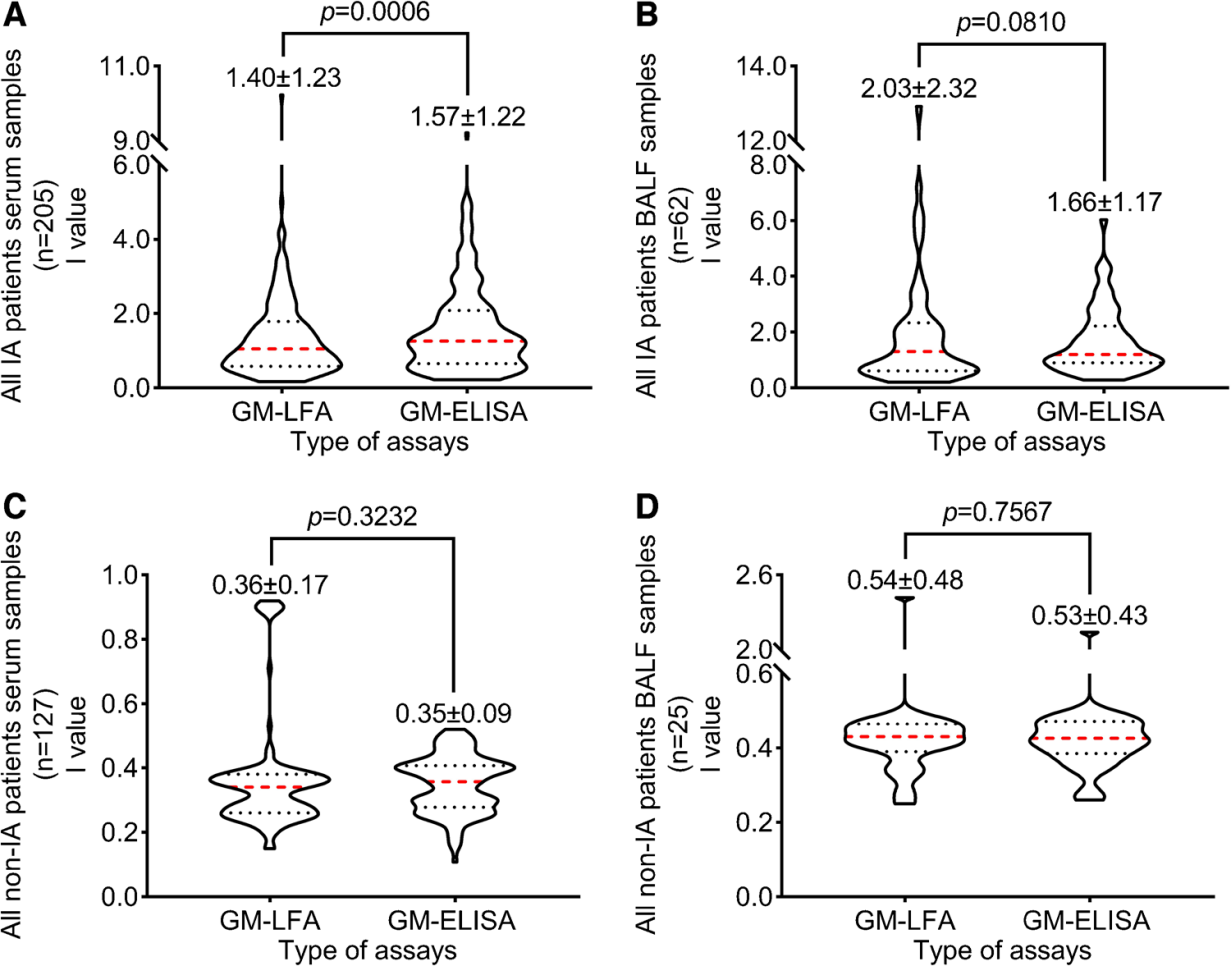
Comparisons of GM *I* values between LFA and ELISA in IA patients and non-IA patients. The mean and standard error are shown.

The median levels detected by LFA in serum samples from the IA group were significantly higher than those detected by ELISA (*P* = 0.006), but there was no significant difference in BALF samples between LFA and ELISA (*P* = 0.081). In the non-IA patient samples, there was no significant difference in serum and BALF samples between LFA and ELISA (*P* = 0.3232 and 0.7567). The detection levels of the two assays are summarized in Fig. 5.

Lymphopenia, neutropenia, and CD4 T cells are essential factors in immunocompromised patients. In the study by Cordonnier et al., there was a significant difference in GM levels between neutropenic patients and nonneutropenic patients [15]. According to some studies, neutropenia is a considerable risk factor for fungal infections.

**Fig. 6:**
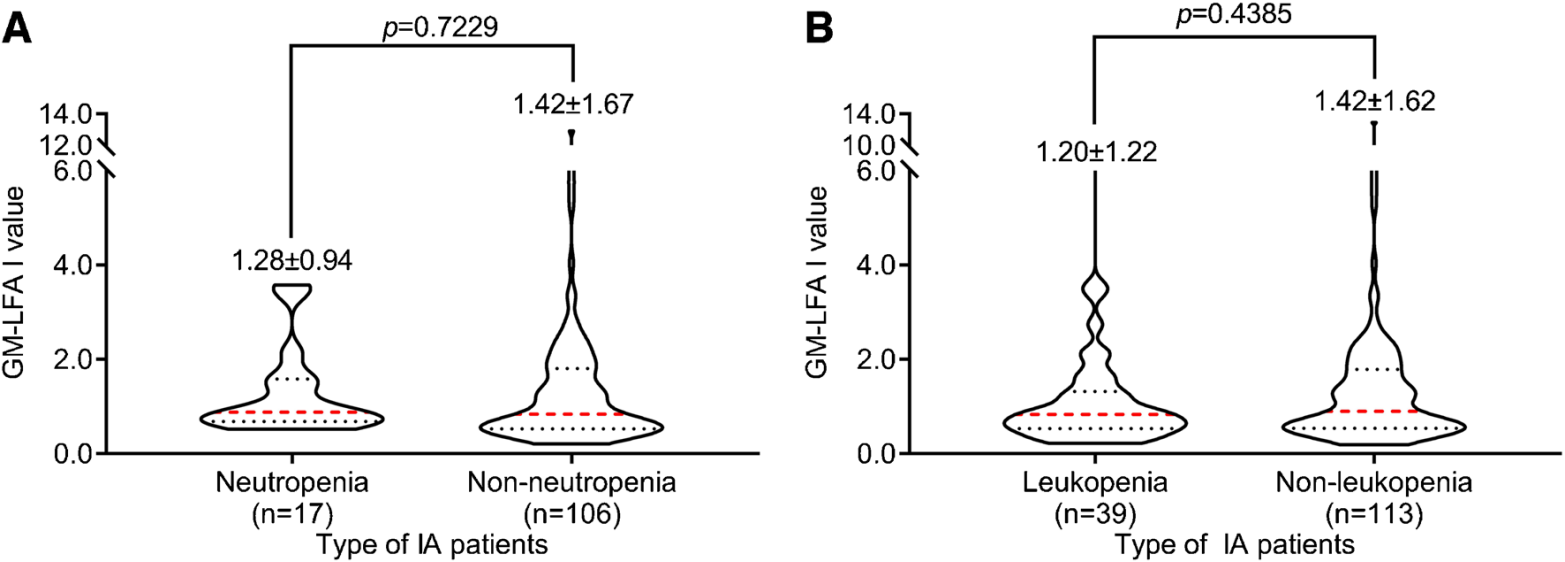
Comparisons of LFA GM *I* values among IA patients with and without neutropenia (**A**). Comparisons of LFA GM *I* values among IA patients with and without leukopenia (**B**). The mean and standard error are shown.

In the study by Susianti et al., there were no significant differences in the total leucocyte count and neutrophil count in the fungal-infected group and nonfungal-infected group [16]. Our results are consistent with the literature in that there were no significant differences in GM *I* values among IA patients with and without neutropenia (*P* > 0.05). There were no significant differences in the GM *I* values in IA patients with and without leukopenia (*P* > 0.05, Fig. 6).

### Performance of LFA and ELISA

**Fig. 7:**
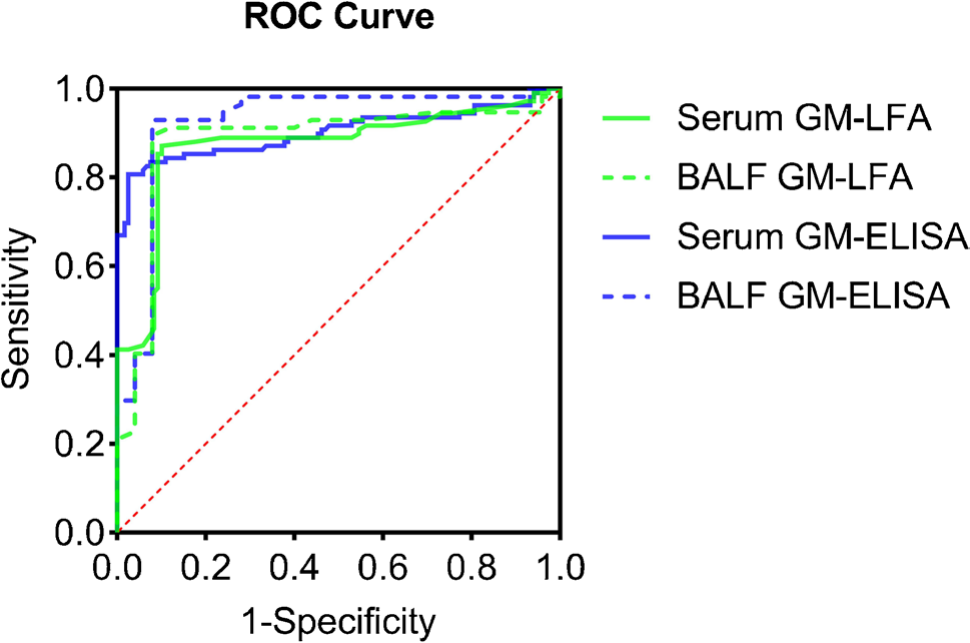
Receiver operating characteristic curves of LFA and ELISA in IA patients and non-IA patients. The AUCs of serum LFA, BALF LFA, serum ELISA, and BALF ELISA were 0.872 (95% CI, 82.14–92.34), 0.882 (95% CI, 79.27–97.08), 0.901 (95% CI, 85.56–94.73), and 0.924 (95% CI, 84.87–99.90), respectively.

At the manufacturer’s recommended GM *I* value of 0.5, the sensitivity and specificity of LFA were 82.57% and 90.76% in serum samples and 89.47% and 92.00% in BALF samples, respectively. The sensitivity and specificity of ELISA in serum samples and BALF samples were greater, as shown in Table 3. ROC analysis confirmed the best LFA *I* values to be 0.4 and 0.5 in serum and BALF samples, respectively (Fig. 7). ROC analysis confirmed the best ELISA optimal positivity threshold to be 0.51 and 0.50 in serum and BALF samples, respectively. The performances of GM (LFA/ELISA) for diagnosing IA at the best *I* value in different samples are shown in Table 4.

**Table 3:** Performance parameters of LFA and ELISA for diagnosing IA at *I* value = 0.5 in serum and BALF

Performance parameters	LFA	ELISA		
	Serum (*n*=228)	BALF (*n*=82)	Serum (*n*=228)	BALF (*n*=82)
Sensitivity (95% CI)	82.57 (73.86–88.92)	89.47 (77.81–95.65)	83.49 (74.89–89.66)	92.98 (82.17–97.73)
Specificity (95% CI)	90.76 (83.70–95.07)	92.00 (72.50–98.60)	92.44 (85.73–96.26)	92.00 (72.50–98.60)
PPV (95% CI)	89.11 (80.96–94.17)	96.23 (85.92–99.34)	91.00 (83.17–95.54)	96.36 (86.39–99.37)
NPV (95% CI)	85.04 (77.37–90.53)	79.31 (59.74–91.29)	85.93 (78.42–91.23)	85.19 (65.39–95.14)

**Table 4:** Performance parameters of LFA and ELISA for diagnosing IA at the best optical density index in serum and BALF

Performance parameters	LFA		ELISA	
	Serum (*n*=228) (ODI=0.40)	BALF (*n*=82) (ODI=0.50)	Serum (*n*=228) (ODI=0.51)	BALF (*n*=82) (ODI=0.50)
Sensitivity (95% CI)	87.16 (79.59–92.19)	89.47 (78.88–95.09)	80.73 (72.34–87.04)	92.98 (83.30–97.24)
Specificity (95% CI)	89.92 (83.20–94.14)	92.00 (75.03–98.58)	97.48 (92.85–99.31)	92.00 (75.03–98.58)
PPV (95% CI)	88.79 (80.86–93.82)	96.23 (85.92–99.34)	96.70 (89.99–99.14)	96.36 (86.39–99.37)
NPV (95% CI)	88.43 (81.03–93.30)	79.31 (59.74–91.29)	84.67 (77.29–90.05)	85.18 (65.39–95.14)

### Kinetic profile analysis of LFA and ELISA

**Fig. 8:**
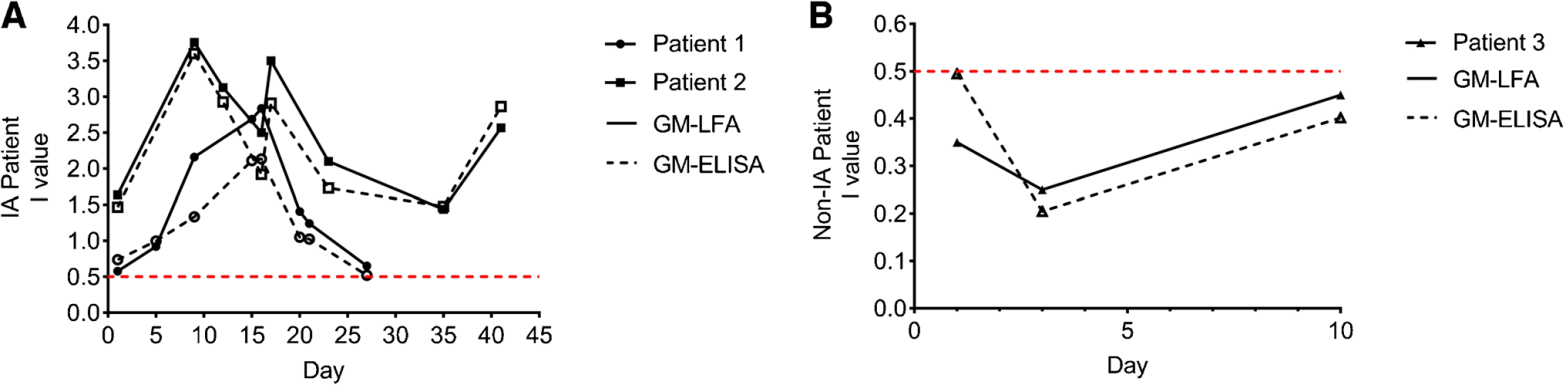
Representative galactomannan (GM) kinetic trends over time for IA patients (**A**) and non-IA patients (**B**) as determined by LFA and ELISA. The red-dotted lines indicate the manufacturer’s recommended optical density index for LFA (*I* value = 0.5) and ELISA (*I* value = 0.5).

We selected three patients and analyzed all of their GM test results. At an *I* value of 0.5, LFA and ELISA could monitor the GM levels in the serum of IA patients and non-IA patients (Fig. 8). Continuous dynamic monitoring of high-risk patients has value in early diagnosis and confirms that monitoring the dynamic change in serum GM content is also conducive to the judgment of the treatment effect and the development of the disease.

## Discussion

In this study, the performance of a galactomannan antigen kit (LFA) with an associated automated fluorescence immunoassay analyzer was evaluated on BALF and serum samples for the diagnosis of IA. The patients were mainly from the respiratory, ICU, and hematology departments. The LFA is considered an important innovation in the mycological sciences. In particular, the usefulness of the test and its early results, high sensitivity and specificity, and ability to work in body fluids other than BALF or serum are important advantages. Some studies have reported on the practicality of the IMMY *Aspergillus* LFA in serum or BALF [10, 17, 18].

The diagnostic performance of LFA and ELISA correlated well in our study. In our analysis, with an optimized cutoff of 0.5 as recommended by the manufacturer, the sensitivity of LFA for the diagnosis of IA in serum samples was 82.57%, the specificity was 90.76%, the PPA was 89.11%, and the NPA was 85.04%. These findings are consistent with the previously published results by Serin et al., in which LFA had a sensitivity of 90.9% and a specificity of 90.8% for an *I* value of 0.5 in predicting IA [19]. In the study by Almeida-Paes et al., the sensitivity of LFA in serum was less than that of ELISA (74% versus 89%) [20]. In a study by Linder et al., BALF GM LFA had poor sensitivity but very high specificity for the diagnosis of IA [21]. We also observed that both ELISA and LFA GM in BALF samples had high specificity for the diagnosis of IA (92.00% versus 92.00%).

In the study by White et al., a qualitative agreement between the IMMY LFA and Bio-Rad ELISA was excellent in case and control populations [22]. In our study, the qualitative agreement between the LFA and ELISA was excellent in both serum and BALF samples. In serum samples, the PPA between the GM LFA and ELISA was 95.11% better than that in BALF samples (93.33%). In BALF samples, the NPA between LFA and ELISA was 96.30%, which was better than that in serum samples (89.19). The semiquantitative correlation between the GM *I* value for case-based samples was excellent (Spearman’s coefficient, *r* = 0.9022).

Other sources of infection could not be excluded from our subjects, so it was difficult to find patients with only fungal infections. Therefore, our study showed that many fungal-infected patients had leukocytosis and neutrophilia. There were no significant differences in GM levels in patients with and without neutropenia. The number of neutropenia samples was rare, so there are some limitations in this study. Hence, our results indicate that BALF is superior to serum in the detection of IA by the ELISA/LFA method. Monitoring the GM levels in IA patients undergoing antifungal treatment showed that GM showed a gradual decrease in patients in remission. The LFA and ELISA results were consistent. These results are consistent with a previous report documenting that GM levels could be used to monitor treatment response in patients with IA[23].

In conclusion, the Dynamiker LFA provides a comparable alternative to ELISA when testing serum and BALF samples. When using the usual 0.5 threshold, the LFA performance was slightly inferior to that of the ELISA companion assay. We found that the BALF *Aspergillus* GM LFA had high sensitivity and specificity for the diagnosis of IA. The test system was simple to use, and the results were highly reproducible.

## Data Availability

The data sets generated during the current study are available from the corresponding authors upon reasonable request.
